# T cell LFA-1-induced proinflammatory mRNA stabilization is mediated by the p38 pathway kinase MK2 in a process regulated by hnRNPs C, H1 and K

**DOI:** 10.1371/journal.pone.0201103

**Published:** 2018-07-26

**Authors:** Gautham K. Rao, Albert Wong, Mark Collinge, Joseph Sarhan, Timur O. Yarovinsky, Vinod S. Ramgolam, Matthias Gaestel, Ruggero Pardi, Jeffrey R. Bender

**Affiliations:** 1 Department of Internal Medicine, Section of Cardiovascular Medicine, Cardiovascular Research Center, Yale University School of Medicine, New Haven, Connecticut, United States of America; 2 Department of Immunobiology, Yale University School of Medicine, New Haven, Connecticut, United States of America; 3 Raymond and Beverly Sackler Foundation Cardiovascular Laboratory, New Haven, Connecticut, United States of America; 4 Department of Cell Biology, Yale University School of Medicine, New Haven, Connecticut, United States of America; 5 Institute of Biochemistry, Medical School Hannover, Hannover, Germany; 6 Faculty of Medicine and Surgery, Università Vita-Salute San Raffaele, Milan, Italy; Ludwig-Maximilians-Universitat Munchen, GERMANY

## Abstract

Activation of the β_2_ integrin lymphocyte function-associated antigen-1 (LFA-1) in T cells induces stabilization of proinflammatory AU-rich element (ARE)-bearing mRNAs, by triggering the nuclear-to-cytoplasmic translocation of the mRNA-binding and -stabilizing protein HuR. However, the mechanism by which LFA-1 engagement controls HuR localization is not known. Here, we identify and characterize four key regulators of LFA-1-induced changes in HuR activity: the p38 pathway kinase MK2 and the constitutive nuclear proteins hnRNPs C, H1 and K. LFA-1 engagement results in rapid, sequential activation of p38 and MK2. Post-LFA-1 activation, MK2 inducibly associates with both hnRNPC and HuR, resulting in the dissociation of HuR from hnRNPs C, H1 and K. Freed from the three hnRNPs, HuR translocates from the nucleus to the cytoplasm, and mediates the stabilization of labile cytokine transcripts. Our results suggest that the modulation of T cell cytokine mRNA half-life is an intricate process that is negatively regulated by hnRNPs C, H1 and K and requires MK2 as a critical activator.

## Introduction

Integrin receptor engagement is essential for leukocyte extravasation at sites of infection and inflammation. In particular, β_2_ integrins play key roles in forming immunological synapses and macromolecular complexes consisting of both structural and signaling proteins. The α_L_β_2_ (CD11a/CD18) integrin lymphocyte function-associated antigen-1 (LFA-1) is expressed in all cells of the hematopoietic lineage [1,2]. LFA-1 is involved in cell adhesion, locomotion and extravasation [3]. During T cell activation, engagement of the T cell receptor/CD3 induces an allosteric transition in LFA-1 (inside-out signaling), resulting in a high-affinity state for its ligand, intercellular adhesion molecule-1 (ICAM-1) [4]. Upon binding to ICAM-1, LFA-1 transduces signaling cascades of its own (outside-in signaling) that result in significant changes in cell motility, cytoskeletal organization, and expression of proinflammatory cytokine genes.

We have previously shown that T cell LFA-1 engagement triggers signaling events that lead to significant stabilization of constitutively labile mRNA transcripts, including TNF-α, IFN-γ, GM-CSF and IL-3, that bear adenylate-uridylate (AU)-rich elements (AREs) in their 3’ untranslated regions (UTRs) [5,6]. We have shown that the mechanism of this LFA-1-induced mRNA stabilization involves the nuclear-to-cytoplasmic translocation of the ubiquitous mRNA-binding and -stabilizing protein, Hu protein R (HuR) [5,6]. The importance of HuR in the stabilization of a variety of labile mRNA transcripts has been widely demonstrated [5,7,8]. Furthermore, the nuclear-to-cytoplasmic translocation of HuR and the proteins that help to effect this translocation have also been described [9,10]. Recent work has further revealed that LFA-1-induced HuR translocation, and consequent cytokine mRNA stabilization, is dependent on a proximal signaling cascade that involves the guanine nucleotide exchange factor, Vav1, the small GTPases, Rac1/2, and mitogen-activated protein (MAP) kinase kinase 3 (MKK3) [6]. However, the distal signaling events downstream of MKK3 that modulate HuR translocation and consequent mRNA stabilization are not completely understood.

MAP kinase-activated protein kinase 2 (MK2), one of the kinases downstream of MKK3, is essential for production of TNFα and IFNγ after exposure to LPS or infection with *Listeria monocytogenes* and has been implicated in modulating mRNA half-life [11–13]. A few groups have further reported a link between activation of the p38 MAP kinase pathway and changes in HuR activity [8,14,15]. These studies, however, have generally focused on the role of p38 and MK2 in regulating mRNA *destabilization*, via post-translational modification (and consequent inactivation) of *trans*-acting destabilization factors [16–19].

A variety of additional proteins has also been shown to associate with HuR in various contexts. Among these are members of the heterogeneous nuclear ribonucleoprotein (hnRNP) family [20], which are known to mediate a wide variety of post-transcriptional regulation, from splicing to transport [21]. While some hnRNPs shuttle between the nucleus and cytoplasm, others, such as hnRNPC, are completely restricted to the nucleus [22,23]. More interestingly, a handful of hnRNP proteins have been implicated in the regulation of mRNA half-life. hnRNPD (AUF-1), for example, is an ARE-binding mRNA destabilization factor that competes with HuR [20].

In this study, we identify four critical regulators of T cell HuR activity downstream of β_2_ integrin activation: MK2 (positive) and hnRNPs C, H1 and K (negative). We show that p38 and MK2 are sequentially activated after LFA-1 engagement. MK2 is required for integrin-induced HuR translocation and consequent stabilization of labile TNF-α and IFN-γ transcripts. Three members of the hnRNP family, hnRNPs C, H1 and K, are constitutively associated with HuR, and dynamically dissociate from HuR upon integrin activation. Upon LFA-1 engagement, MK2 physically associates with both HuR and hnRNPC, triggering hnRNPC to dissociate from HuR (an event which is abrogated in MK2 gene-deleted T cells). HuR (but not hnRNPs C, H1 or K) then translocates to the cytoplasm and associates with, and stabilizes, proinflammatory transcripts. Consistent with a negative regulatory role, if expression of hnRNPC, H1 or K is inhibited, cytokine mRNAs are constitutively stabilized, even without LFA-1 activation. Together, our results demonstrate that LFA-1 engagement activates a p38- and MK2-dependent distal signaling cascade that results in the dissociation of hnRNPs C, H1 and K from HuR and consequent HuR translocation and mRNA stabilization. Our findings suggest that MK2 and hnRNPs C, H1 and K may be suitable targets for the development of novel, specific immunomodulators.

## Results

### LFA-1 engagement activates p38 and MK2, and this activation is required for HuR translocation and mRNA stabilization

The mechanism by which LFA-1 effects changes in HuR localization and function is not known. Due to the requirement for MKK3 [6], and the previously identified role of its downstream kinase MK2 in negatively regulating RNA destabilizing factors [16–19], we addressed whether MK2 positively regulates HuR (and consequent transcript stabilization) in an LFA-1-stimulated manner.

**Fig 1:**
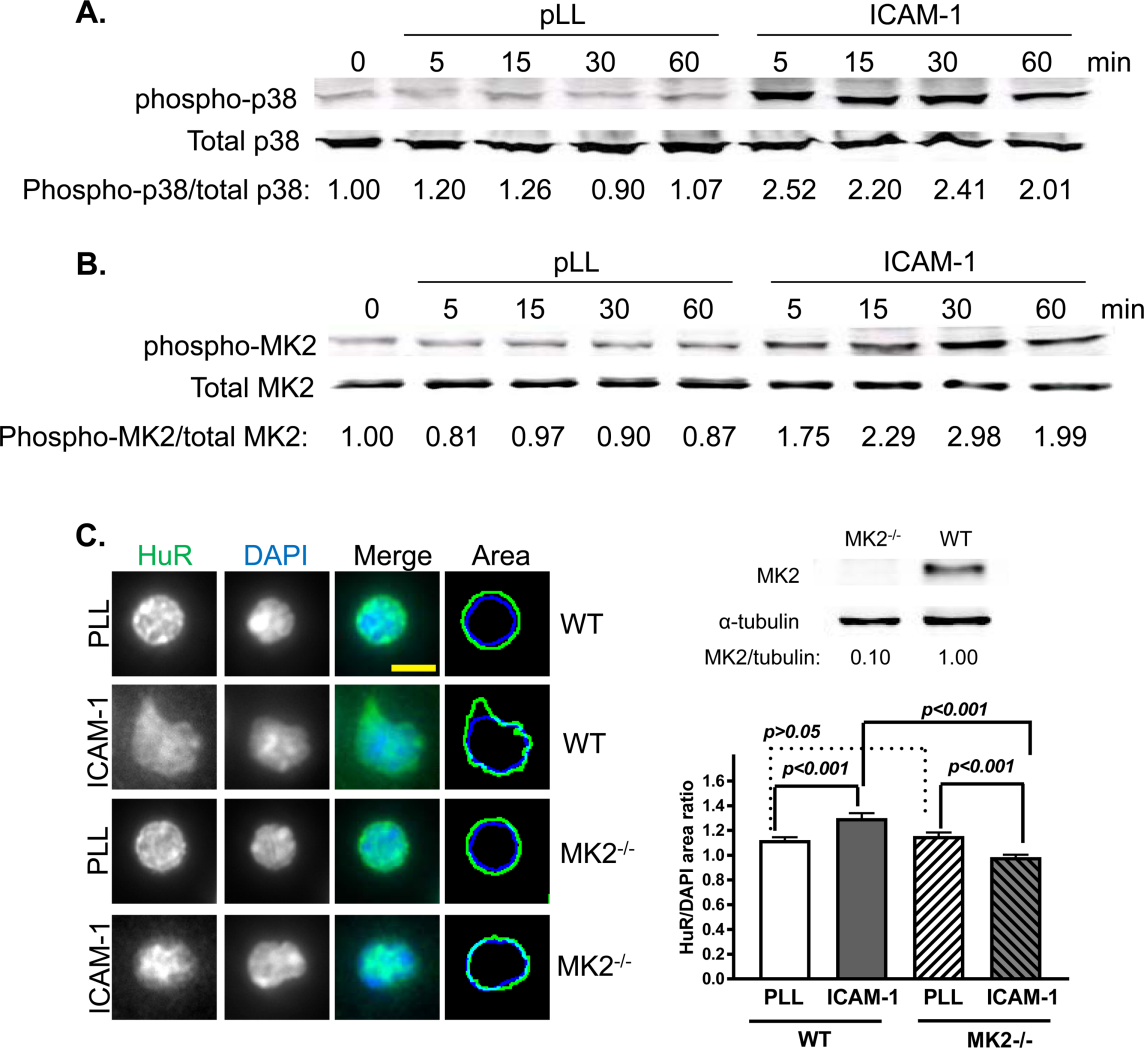
p38 and MK2 are activated upon LFA-1 engagement, and MK2 is required for LFA-1-induced HuR translocation. (**A**, **B**) LFA-1-induced activation of p38 and MK2. Human Jurkat T cells were adhered to pLL- or ICAM-1-coated plates for 5, 15, 30, or 60 min, then lysed. Lysates were subjected to phospho-p38 (**A**) or phospho-MK2 (**B**) immunoblotting. Data are representative of three independent experiments. (**C**) Requirement of MK2 for LFA-1-induced HuR translocation. WT or MK2^-/-^ primary murine T cells were adhered to pLL- or ICAM-1-coated coverslips for 45 min, and subjected to IF using an anti-HuR antibody and the nuclear marker DAPI. Quantification of HuR translocation is shown as a ratio of area within the periphery of HuR staining (green contours) divided by nuclear area (blue contours). Data represent three independent experiments with standard error. Scale bar, 10 μm.

In Western blotting experiments, using lysates from Jurkat T cells adhered to poly-L-lysine (pLL) control or recombinant ICAM-1, we biochemically confirmed a rapid, sustained LFA-1-induced phosphorylation of both p38 and MK2 (Fig 1A and 1B). Translocation of HuR to the cytoplasm is required for, and an indicator of, HuR-mediated mRNA stabilization. If MK2 is a critical effector of LFA-1-induced cytokine transcript stabilization, this should be reflected in its requirement in integrin-triggered HuR translocation. Fig 1C displays the absence of induced HuR nuclear-to-cytoplasmic translocation upon adhesion of MK2 knockout primary murine splenic T cells to ICAM-1, in contrast to that observed in wild-type T cells.

**Fig 2:**
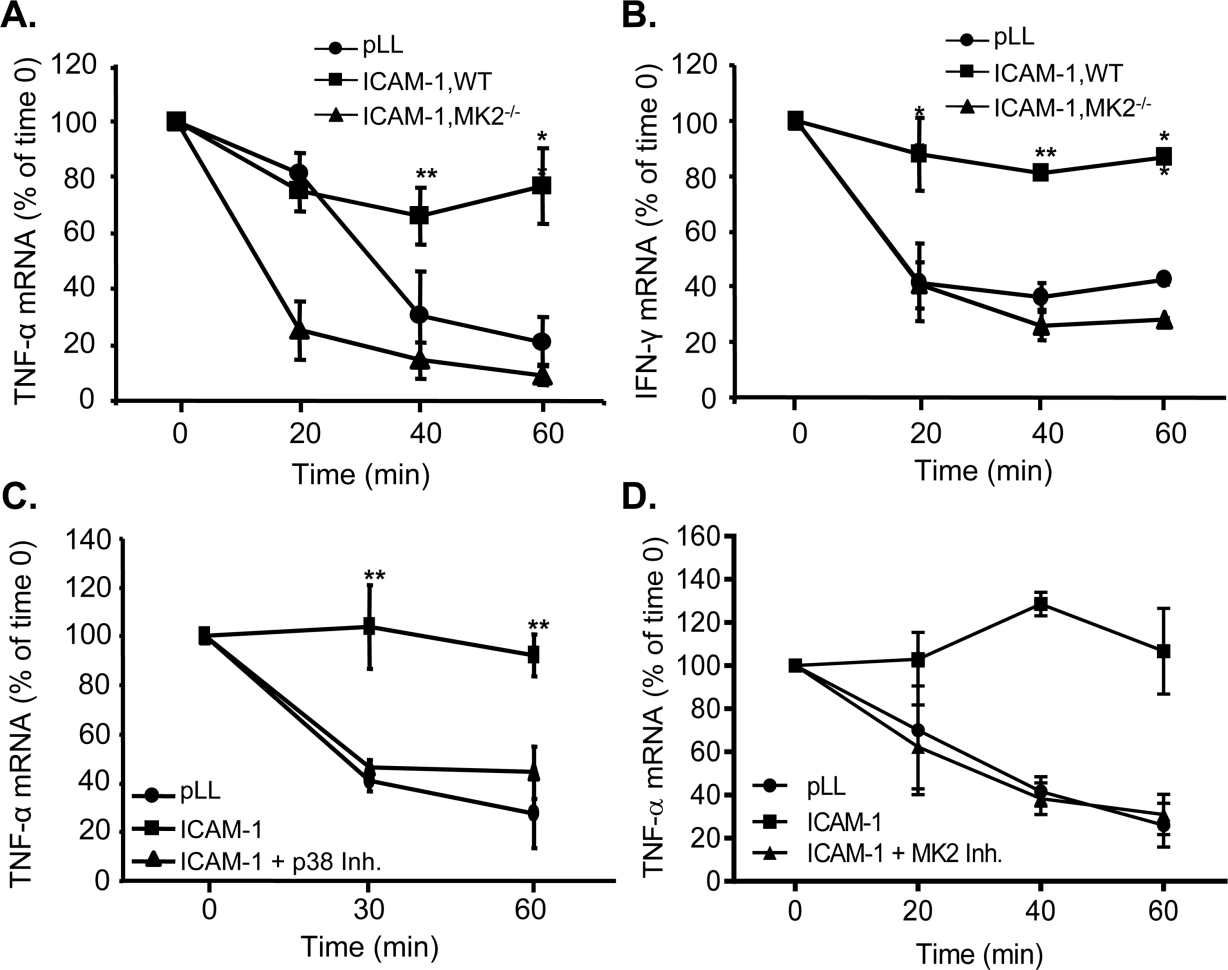
Activated p38 and MK2 are functionally required for LFA-1-induced proinflammatory mRNA stabilization. (**A**, **B**) WT or MK2^-/-^ primary murine T cells were adhered to pLL- or ICAM-1-coated plates, treated with PMA for 3 h to maximize *TNF* and *IFNG* gene transcription, then treated with transcriptional inhibitor DRB and lysed at 0, 20, 40, or 60 min. Total RNA was isolated from the lysates, and TNF-α (**A**) and IFN-γ (**B**) levels at each timepoint, normalized to GAPDH, were determined using qRT-PCR relative to time 0 (set at 1.0) levels. Data represent three independent experiments. (**C, D**) Jurkat T cells were pretreated with p38 inhibitor (C), MK2 inhibitor (D), or DMSO vehicle for 15 min, adhered to pLL- or ICAM-1-coated plates for 30 min, then treated with DRB and lysed at indicated timepoints. Total RNA was isolated from the lysates, and TNF-α mRNA levels at each timepoint, normalized to GAPDH, were determined using qRT-PCR and compared to time 0 levels. Data represent three independent experiments. **, p < 0.01.

The lack of LFA-1-induced HuR translocation in MK2 knockout T cells is predictive of the loss of integrin activation-induced mRNA half-life extension, previously shown to be HuR-dependent [5,6]. Indeed, stabilization of the intrinsically labile proinflammatory mRNAs TNF-α and IFN-γ in LFA-1-engaged wild-type T cells is abrogated in MK2 knockout T cells (Fig 2A and 2B). Likewise, extension of TNF-α mRNA half-life in LFA-1-engaged Jurkat T cells is lost in cells pretreated with either a p38 (Fig 2C) or MK2 inhibitor (Fig 2D). These results demonstrate that LFA-1 engagement activates the p38-MK2 pathway, and that activated MK2 is functionally required for integrin-induced, HuR-dependent cytokine mRNA stabilization.

### hnRNPs C, H1 and K are constitutive, dynamic HuR-associated proteins

Because multiple mRNA-binding proteins are known to interact with HuR in various contexts, we addressed whether any are constitutively bound to HuR in T cells. To identify such HuR-associated proteins, we conducted a high-throughput proteomic screening assay using HuR immunoprecipitate from Jurkat T cell lysates (Table 1) LC-MS/MS analysis revealed a strong match (nine peptides) to hnRNPC1/C2 isoform b. Other proteins identified in our analysis included actin, fibrillarin, and RNA-binding protein Raly isoform 2. Since LC-MS/MS assay of the HuR immunoprecipitates was constrained by the interference from the anti-HuR immunoglobulin heavy and light chains, we extended our proteomics analyses of HuR associated proteins in resting and ICAM-1-stimulated cells using HuR-GST pulldown and isobaric tag for relative and absolute quantitation (iTRAQ) of phosphopeptides (S1 Table). Additional proteins identified from those screens included polyadenylate binding protein, elongation factor 1α, hnRNPs A1, H1 and K, and histone H1.

**Table 1:** MALDI-MS/MS analyses of HuR co-immunoprecipitates from Jurkat cell lysates.

MW range (kDa)	Protein identified	Genbank Accession number	Number of peptides matched
23–25	HuR RNA binding protein	AAB41913.1	2
23–25	tubulin, beta 2C	AAH29529.1	2
28–31	HuR RNA binding protein	AAB41913.1	3
28–31	fibrillarin	AAP35476.1	3
28–31	actin, cytoplasmic 1	NP_001092.1	1
31–33	heterogeneous nuclear ribonucleoproteins C1/C2 isoform b	NP_004491.2	9
31–33	RNA-binding protein Raly isoform 2	NP_031393.2	2
33–35	actin, cytoplasmic 1	NP_001092.1	6
35–40	actin, cytoplasmic 1	NP_001092.1	9

**Fig 3:**
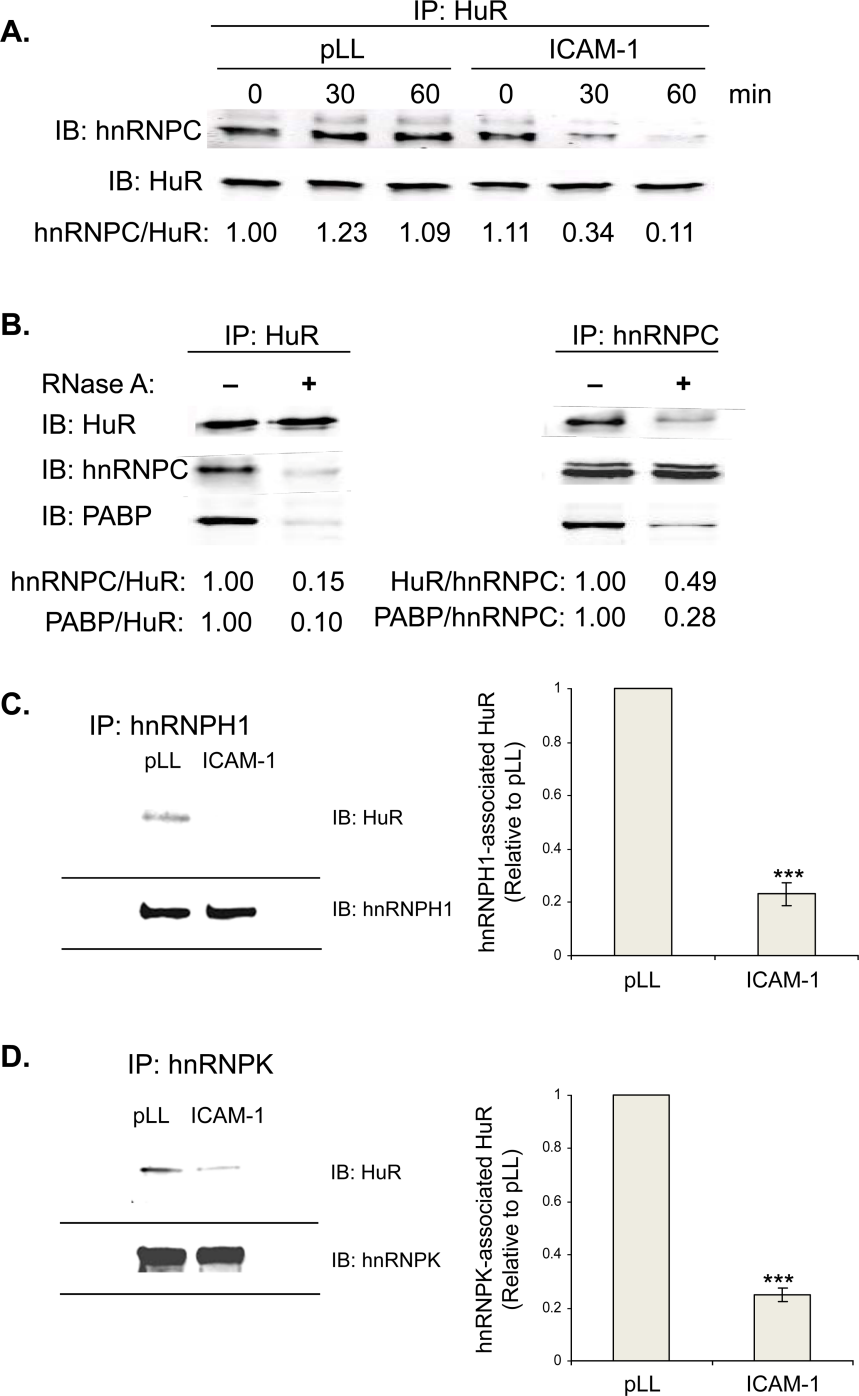
hnRNPC constitutively associates with HuR, but inducibly dissociates upon LFA-1 engagement. (**A**) LFA-1-induced dissociation of HuR and hnRNPC. Jurkat T cells were adhered to pLL- or ICAM-1-coated plates for 0, 30, or 60 min, then lysed. Lysates were subjected to HuR IP, followed by hnRNPC blotting. Data is representative of three independent experiments. (**B**) RNA dependence of HuR-hnRNPC association. Jurkat T cells were lysed and subjected to RNase A treatment for 30 min, then subjected to HuR or hnRNPC IP, followed by HuR, hnRNPC and PABP blotting. Data is representative of three independent experiments. (**C, D**) LFA-1-induced dissociation of hnRNPs H1 and K from HuR. Primary human T cells were adhered to pLL- or ICAM-1-coated plates for 30 min, then lysed and subjected to hnRNPH1 (**C**) or hnRNPK (**D**) IP, followed by HuR blotting. Densitometric quantification of hnRNPH1 or hnRNPK associated with HuR is shown. Data represent three independent experiments. ***, p < 0.001.

In co-immunoprecipitation experiments using lysates from Jurkat T cells adhered to pLL control, we biochemically confirmed a constitutive, RNA-stabilized hnRNPC-HuR association (Fig 3A and 3B). LFA-1 activation by T cell adhesion to ICAM-1 resulted in rapid, significant hnRNPC-HuR dissociation, establishing the integrin-regulated dynamic nature of this interaction (Fig 3A). Association of HuR with hnRNPC and poly(A) tail-binding protein PABP was markedly reduced by RNase A treatment, suggesting that RNA stabilizes the HuR-hnRNPC-PABP complex (Fig 3B). Similar LFA-1-regulated dynamic interactions were observed in primary human peripheral blood T cells between hnRNPH1 and HuR, and hnRNPK and HuR (Fig 3C and 3D).

### MK2 associates with both hnRNPC and HuR upon LFA-1 engagement, triggering hnRNPC-HuR dissociation

**Fig 4:**
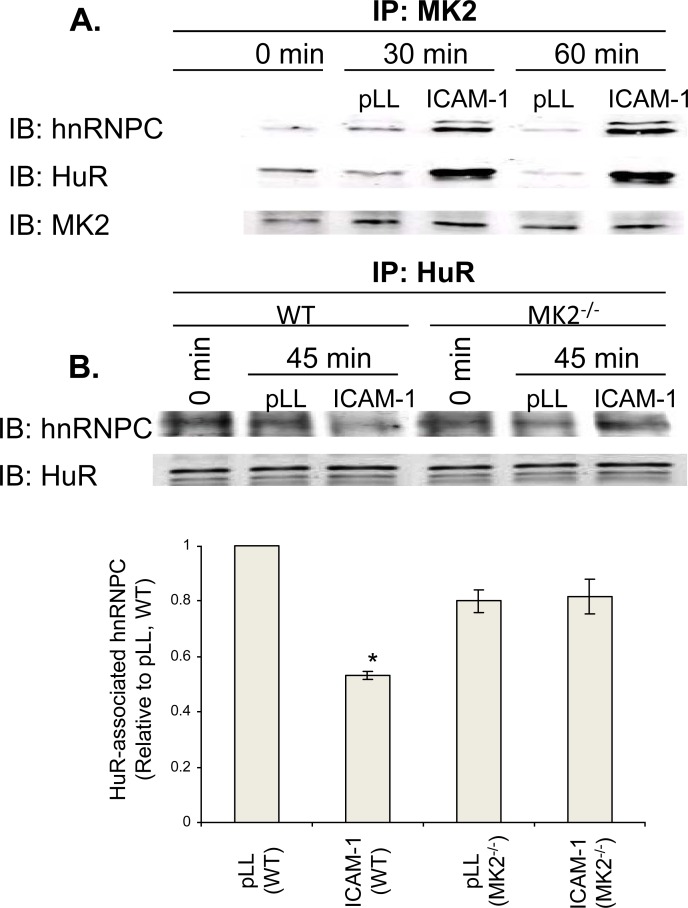
MK2 inducibly associates with both hnRNPC and HuR, triggering hnRNPC-HuR dissociation. (**A**) LFA-1-induced association of MK2 with hnRNPC and HuR. Jurkat T cells were adhered to pLL- or ICAM-1-coated plates for 0, 30, or 60 min, then lysed. Lysates were subjected to MK2 IP, followed by hnRNPC and HuR blotting. Data is representative of three independent experiments. (**B**) Requirement of MK2 for LFA-1-induced hnRNPC-HuR dissociation. WT or MK2^-/-^ primary murine T cells were adhered to pLL- or ICAM-1-coated plates for 45 min, then lysed. Lysates were subjected to HuR IP, followed by hnRNPC blotting. Densitometric quantification of hnRNPC associated with HuR is shown, with pLL level set at 1.0. Data is representative of three independent experiments. *, p < 0.05.

As we have determined that MK2 is a critical downstream effector of LFA-1-induced changes in HuR activity, we addressed whether MK2 itself interacts with HuR in the context of integrin activation. Indeed, in co-immunoprecipitation experiments, we observed a rapid, LFA-1-induced (ICAM-1) MK2-HuR association (Fig 4A). As hnRNPC and HuR are constitutively associated, we likewise detected an induced MK2-hnRNPC association (Fig 4A). Significantly, hnRNPC-HuR dissociation in LFA-1-engaged wild-type T cells is abrogated in MK2 knockout T cells (Fig 4B), suggesting that MK2 may regulate HuR translocation and mRNA-stabilizing activity by triggering its dissociation/release from hnRNPC.

### hnRNPs C, H1 and K are basal HuR negative regulators

**Fig 5:**
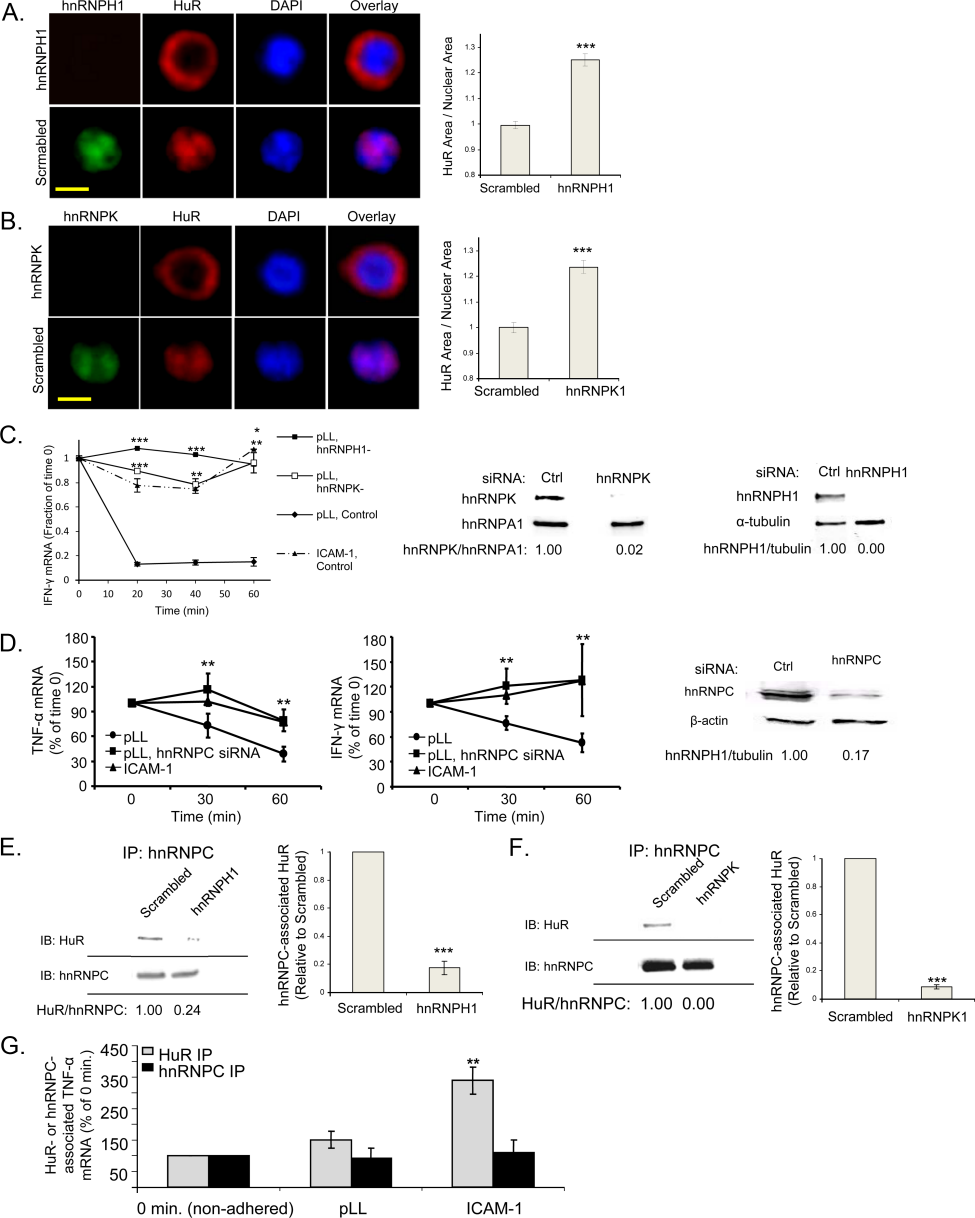
hnRNPs C, H1 and K constitutively associate with, but negatively regulate, HuR at baseline. (**A, B**) Baseline HuR localization in absence of hnRNPs H1 or K. Jurkat T cells were transfected with control siRNA or siRNA against hnRNPH1 (**A**) or hnRNPK (**B**). Cells were subjected to IF using an anti-HuR antibody and the nuclear marker DAPI. Quantification of HuR translocation is shown. Data represent four independent experiments. Scale bar, 10 μm. ***, p < 0.001 (**C**) Effect of absence of hnRNPs H1 or K on mRNA stability. Jurkat T cells were transfected with control siRNA or siRNA against hnRNPH1 or hnRNPK. Cells were adhered to pLL- or ICAM-1-coated plates for 30 min, then treated with DRB and lysed at 0, 20, 40, or 60 min. Total RNA was isolated from the lysates, and IFN-γ mRNA levels at each timepoint, normalized to GAPDH, were determined using qRT-PCR and compared to time 0 levels. Data represent three independent experiments. *, p < 0.05; **, p < 0.01; ***, p < 0.001 (pLL, hnRNPH1– or pLL, hnRNPK–vs. pLL, Control) (**D**) Effect of absence of hnRNPC on mRNA stability. Jurkat T cells were transfected with control siRNA or siRNA against hnRNPC. Cells were adhered to pLL- or ICAM-1-coated plates for 30 min, then treated with DRB and lysed at 0, 30, or 60 min. Total RNA was isolated from the lysates, and TNF-α and IFN-γ mRNA levels at each timepoint, normalized to GAPDH, were determined using qRT-PCR and compared to time 0 levels. Data represent five independent experiments. **, p < 0.01 (**E, F**) Requirement of hnRNPs K and H1 for sequestration of HuR and hnRNPA1 to hnRNPC at baseline. Jurkat T cells were transfected with control siRNA or siRNA against hnRNPH1 (**E**) or hnRNPK (**F**). Cells were lysed and subjected to hnRNPC IP, followed by HuR and hnRNPA1 blotting. Densitometric quantification of HuR or hnRNPA1 associated with hnRNPC is shown. Data represent three independent experiments. ***, p < 0.001 (**G**) LFA-1-induced association of HuR with cytokine transcripts. Jurkat T cells were adhered to pLL- or ICAM-1-coated plates for 45 min, fixed with formaldehyde, then lysed. Lysates were subjected to HuR or hnRNPC IP, followed by reversal of crosslinking. Total RNA was isolated from the immunoprecipitates, and TNF-α mRNA levels, normalized to 18s rRNA, were determined using qRT-PCR and compared to non-adhered (time 0, set at 1.0) levels. Data represent five independent experiments.

We postulated that the constitutive, basal association of hnRNPs C, H1 and K with HuR negatively regulates HuR by sequestering it in the nucleus, blocking its ability to translocate and bind to labile, ARE-bearing mRNA transcripts. To test this, HuR subcellular localization was assessed in pLL-adhered, siRNA-mediated hnRNPH1 or K knockdown Jurkat T cells. Fig 5A and 5B immunofluorescent HuR micrographs indeed display HuR’s cytoplasmic localization in the knockdown cells, identical to that seen in LFA-1-stimulated T cells. Additionally, as we typically observe when HuR is cytoplasm-localized, the labile IFN-γ transcript is remarkably stable in transcription-arrested, pLL-adhered, hnRNPH1 or K knockdown T cells, similar to the stabilization induced by LFA-1 engagement (Fig 5C). Similar results were observed in hnRNPC knockdown T cells as well (Fig 5D). Further supporting our nuclear sequestration model, siRNA-mediated knockdown of either hnRNPH1 or hnRNPK is sufficient to disrupt HuR’s basal association with hnRNPC (Figs 5E and 4F). Treatment with any of the siRNAs (hnRNPC, hnRNPH1, hnRNPK-specific or the corresponding scrambling controls) did not cause any cell aggregation or adhesion to ICAM-1, indicating lack of LFA-1 activation. Finally, to confirm that the negative, basal hnRNP regulation of HuR is not a result of competitive inhibition of HuR binding to mRNA, we obtained HuR or hnRNPC immunoprecipitate from pLL control- or ICAM-1-adhered Jurkat T cells subjected to formaldehyde-induced protein-RNA crosslinking. Consistent with previous reports of HuR regulation of mRNA stability through binding to the 3’ UTR [24], we observed a significant increase in HuR-associated TNF-α mRNA in LFA-1-engaged T cells (Fig 5G). In contrast, there was no LFA-1-induced decrease in hnRNPC-associated TNF-α transcripts (Fig 5G). Taken together, these findings demonstrate that hnRNPs C, H1 and K serve as critical basal regulators of T cell mRNA half-life, promoting maintenance of HuR in its “inactive” form.

## Discussion

Engagement of T cell LFA-1 results in a significant stabilization of normally labile proinflammatory transcripts, including those encoding TNF-α, IFN-γ, GM-CSF and IL-3 [5,6]. We have previously shown that this integrin-induced mRNA stabilization, and consequent increase in protein expression, is dependent on the nuclear-to-cytoplasmic translocation of HuR [5,6]. However, the mechanism by which LFA-1 controls HuR localization and activity has remained elusive. We now show, for the first time, that HuR activity and localization is regulated downstream of LFA-1 engagement by MK2 and hnRNPC. p38 and MK2 are rapidly and sequentially activated after integrin engagement. MK2 inducibly associates with both HuR and hnRNPC, triggering hnRNPC-HuR dissociation and consequent HuR translocation and transcript stabilization. In the absence of LFA-1 activation, HuR is negatively regulated through its hnRNPC association, blocking HuR binding to mRNA transcripts.

MK2 and hnRNPs C, H1 and K are ubiquitously expressed proteins with roles in a broad array of cellular signaling processes, including ones relevant to transcription and post-transcriptional processing [7,8,21,25]. In particular, multiple hnRNP family members have been shown to bind to and regulate mRNAs, including modulation of mRNA half-life [26–28]. However, our study focuses on the HuR-interacting nature of hnRNPs C, H1 and K, and how these interactions affect HuR’s mRNA-binding and -stabilizing function. The lack of hnRNP C, H1 or K relocalization after LFA-1 engagement, coupled with our observation that there is no integrin-induced alteration in hnRNPC’s association with cytokine transcripts, reaffirm our previous conclusion that HuR is the key effector of T cell β_2_ integrin-induced changes in cytokine mRNA stability [5,6].

p38 and MK2 have been shown to post-translationally modify (via phosphorylation) a number of different proteins involved in RNA regulation [16–19]. We do not exclude an important role of MK2 kinase activity in any of these processes in the context of T cell activation. However, we were unable to identify an LFA-1-induced, MK2-mediated phosphorylation of either HuR or hnRNPs C, H1 or K. It is possible that the integrin-triggered physical association of MK2 with the basal HuR-hnRNP complex is sufficient to affect the stability of the complex, allowing HuR to dissociate and leave the nucleus.

Our results indicating an RNA dependence of the basal HuR-hnRNP complex suggest that HuR does not associate with the complex in isolation, but rather in the context of a larger protein complex bound to RNA. Although we cannot rule out direct protein-protein interactions, the basal association of HuR with hnRNPC is likely due to binding to the same transcripts. Our observation that the mRNA poly(A) tail-binding protein PABP co-immunoprecipitates with both HuR and hnRNPC lends further support to the existence of an intricate, basal protein-RNA complex. Using the publicly available databases with experimentally validated crosslinking immunoprecipitation (CLIP) data on protein-RNA interactions, we found that most of the hnRNPC target transcripts also contain binding sites for HuR. Interestingly, many HuR and hnRNPC binding sites are found in the intronic sequences, suggesting that both RNA binding proteins may participate in processing and splicing of constitutively expressed mRNAs.

Under our experimental conditions, T cell LFA-1 engagement triggers dramatic translocation of HuR, from the nucleus to the cytoplasm. However, even lesser amounts of HuR relocalization may be sufficient to achieve the same level of mRNA stabilization observed in LFA-1-activated cells. Unlike knockdown of hnRNPH1 or K, knockdown of hnRNPC results in only a moderate amount of HuR translocation, in comparison to that seen in LFA-1-activated cells. Nevertheless, hnRNPC knockdown is sufficient to mimic the effect of integrin engagement on cytokine mRNA half-life, as we observed for both TNF-α and IFN-γ transcripts. This may indicate that LFA-1-induced proinflammatory mRNA stabilization is a binary, all-or-none switch, rather than a graded response.

**Fig 6:**
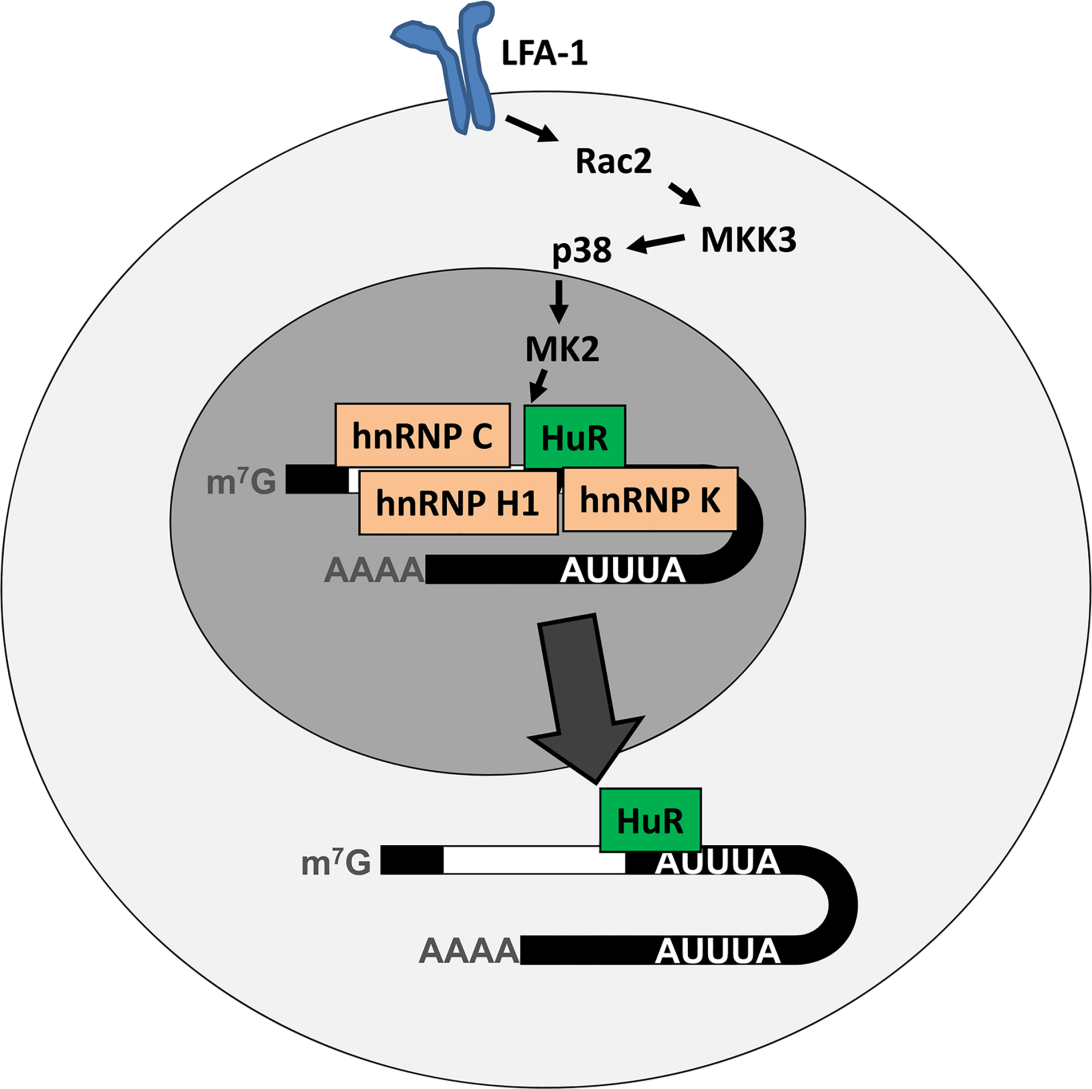
Molecular pathways from LFA-1 to HuR translocation and mRNA stabilization. MK2 activation results in dissociation of nuclear RNA-dependent complex of HuR with hnRNP C, K, and H1 and translocation of HuR to cytoplasm leading to stabilization of cytokine mRNAs.

This is the first report, to our knowledge, to demonstrate the involvement of hnRNPs C, H1 and K in LFA-1-induced mRNA stabilization. Moreover, the present studies are also the first to show the association of MK2 with both hnRNPC and HuR, particularly downstream of LFA-1 engagement. A previous report has implicated MK2 in HuR-dependent mRNA stabilization [8], but without clear indication of the mechanisms involved. Our present model (Fig 6) proposing the involvement of MK2 in the LFA-1-mediated dissociation of HuR from hnRNPs C, H1 and K is, therefore, both novel and unique. The focus on p38 and MK2 in the induction of HuR-dependent stabilization, rather in the modulation of destabilization factors adds additional novelty to the present work. The present studies clearly implicate a positive role for MK2 and negative role for hnRNPs C, H1 and K in LFA-1-induced HuR-dependent mRNA stabilization. Further work is needed to elucidate the complex mechanism by which MK2 and hnRNPs C, H1 and K modulate HuR activity, and studies are actively ongoing to investigate just such a mechanism.

## Materials and methods

### Ethics statement

The Yale University Human Research Protection Program approved all protocols and experiments involving isolation of PBMC from human subjects. The Yale University Institutional Animal Care and Use Committee approved all experimental animal procedures including euthanasia by CO_2_ inhalation.

### Cell culture and reagents

Jurkat T cells were obtained from ATCC (Manassas, VA) and cultured in RPMI 1640 supplemented with 10% FBS and 2 mM L-glutamine. Mouse monoclonal anti-hnRNPC (clones 4F4 and EP3034Y), and rabbit polyclonal hnRNPH1 and PABP antibodies were from Abcam (Cambridge, MA). iScript cDNA synthesis kit and QuantiTect SYBR Green PCR kit were from Bio-Rad (Hercules, CA). Rabbit polyclonal anti-phospho-p38 (Thr-180/Tyr-182), phospho-MK2 (Thr-334), p38 and MK2 antibodies were from Cell Signaling (Danvers, MA). SB203580 (p38 inhibitor) was from EMD Millipore (Darmstadt, Germany). cOmplete mini protease inhibitor was from Hoffman-La Roche (Basel, Switzerland). Fcγ-specific goat-anti human IgG was from Jackson ImmunoResearch (West Grove, PA). Pan T cell isolation kit (II) was from Miltenyi Biotec (San Diego, CA). RNeasy mini kit was from QIAGEN (Hilden, Germany). Recombinant (r) human and mouse ICAM-1 were from R&D (Minneapolis, MN). Goat anti-actin (I-19), mouse monoclonal anti-HuR (clone 3A2) and hnRNPK (clone 3C2) antibodies were from Santa Cruz (Dallas, TX). 4’,6-diamidino-2-phenylindole (DAPI), 5,6-dichloro-1-β-D-ribofuranosyl-1H-benzimidazole (DRB), Histopaque-1077, pLL and phorbol 12-myristate 13-acetate (PMA) were from Sigma-Aldrich (St. Louis, MO). Mouse T cell enrichment kit was from STEMCELL (Vancouver, Canada). SYPRO ruby protein gel stain was from Thermo Fisher (Waltham, MA).

The following primers were synthesized by the W.M. Keck Biotechnology Resource Laboratory (Yale University, New Haven, CT): human 18s sense: 5’-CGCGGTTCTATTTTGTTGGTTT-3’; human 18s antisense: 5’-GCGCCGGTCCAAGAATTT-3’; human GAPDH sense: 5’-ACCAGCCCCAGCAAGAGCACAAG-3’; human GAPDH antisense: 5’-TTCAAGGGGTCTACATGGCAACTG-3’; human IFN-γ sense: 5’-GTCGCCAGCAGCTAAAACAGG-3’; human IFN-γ antisense: 5’-TGCAGGCAGGACAACCATTACT-3’; human TNF-α sense: 5’-GACAAGCCTGTAGCCCATGT-3’; human TNF-α antisense: 5’-TTGATGGCAGAGAGGAGGTT-3’; mouse GAPDH sense: 5’-AACTTTGGCATTGTGGAAGG-3’; mouse GAPDH antisense: 5’-ACACATTGGGGGTAGGAACA-3’; mouse IFN-γ sense: 5’-AGCGGCTGACTGAACTCAGATTGTAG-3’; mouse IFN-γ antisense: 5’-GTCACAGTTTTCAGCTGTATAGGG-3’; mouse TNF-α sense: 5’-CACGTCGTAGCAAACCACCAA-3’; mouse TNF-α antisense: 5’-AGCAAATCGGCTGACGGTGT-3’.

### Primary T cell isolation

Peripheral blood samples were collected from healthy adult donors with written informed consent pursuant to Yale University IRB-approved guidelines. Peripheral blood mononuclear cells (PBMCs) were obtained from heparinized blood diluted three-fold with RPMI-1640 medium by centrifugation over Ficoll-Paque^TM^ PLUS gradient medium (1.077 g/ml, GE Healthcare). T cells were isolated from the PBMCs by negative selection using the pan T cell isolation kit.

### Mice

MK2^-/-^ mice on a C57Bl/6 (H-2^b^) background were generated as described [11]. The MK2^-/-^ mice were viable and fertile with no developmental deficiencies and normal immune cell profiles [13]. C57Bl/6 mice were from Jackson Laboratory (Bar Harbor, ME). Splenic T cells from C57Bl/6 and MK2^-/-^ mice were isolated by negative selection using the mouse T cell enrichment kit. T cell purity was assessed by flow cytometry (CD3+ > 95%). All animal experiments were performed in accordance with Yale University IACUC-approved protocols.

### siRNA and transfection

The following siRNAs were custom synthesized by QIAGEN: hnRNPC: 5’-aaUGAAGAAAGAUGAGACUAA-3’; scrambled control: 5’-aaGAGAAACGAAAUUAGAGUA-3’. Silencer® Select siRNAs for hnRNPH1 (s6730, 5’-GGUAAAACUUAGAUGUCCUTT-3’) and hnRNPK (s6738, 5’-GGGUGUGAUCCAAGCUAUCTT-3’) were from Thermo Fisher (Waltham, MA).

siRNA transfection was performed with 400 nM (hnRNPC or hnRNPK) or 50 nM (hnRNPH1) siRNA, or 400 or 50 nM scrambled control siRNA, via electroporation at 500 μF, 0.40 kV with the Gene Pulser system (Bio-Rad). Jurkat cells (10^7^ cells/transfection) were washed and resuspended in 500 μl Opti-MEM immediately prior to electroporation. After transfection, cells were transferred to 2.5 ml normal culture medium and allowed to recover at 37°C for 24 h. Cells were then added to 7 ml fresh medium and allowed to proliferate for another 24 h. Viable cells were recovered by centrifugation over a Histopaque-1077 cushion, and subjected to a second round of electroporation and recovery.

### mRNA stability assay

Petri dishes were coated with goat anti-human IgG (10 μg/ml) in 50 mM Tris, pH 9.5 for 1 h, blocked with calcium- and magnesium-free (CMF)-PBS containing 2% dialyzed FBS for 1 h, then incubated with rICAM-1 (100 ng/ml) overnight at 4°C. Control dishes were coated with poly-L-lysine (0.005%) overnight at 4°C. T cells (3.5*10^6^ Jurkat cells/group or 5*10^6^ primary murine T cells/group) were washed and resuspended in CMF-PBS (control) or LFA-1 activation buffer (100 mM Tris, pH 7.5, 150 mM NaCl, 2 mM MgCl_2_, 2 mM MnCl_2_, 5 mM D-glucose, 1.5% BSA), and allowed to adhere to the coated Petri dishes for 30 min at 37°C. For p38 or MK2 inhibition experiments, cells were additionally pretreated with p38 inhibitor (10 μM), or an equal volume of DMSO (control, 0.1% v/v), for 15 min at 37°C after resuspension in CMF-PBS or LFA-1 activation buffer but prior to adhesion. Transcription was blocked with the addition of DRB (250 μM). For experiments with primary murine T cells, mRNA transcription was stimulated with PMA (10 ng/ml) for 3 h at 37°C prior to DRB addition. Cells were lysed at various time points after DRB addition (between 0–60 min) in Buffer RLT, and total RNA was isolated using the RNeasy mini kit. 1 μg total RNA was reverse transcribed using the iScript cDNA synthesis kit, and subjected to real-time PCR analysis using the QuantiTect SYBR Green PCR kit with the Opticon DNA Engine 2 (Bio-Rad). Samples were run in duplicate using the following cycling parameters: 95°C for 15 min, then 50 cycles of 95°C for 30 sec, 56°C for 60 sec, and 72°C for 60 sec. mRNA levels were normalized to GAPDH to control for loading.

### Western blot analysis

Petri dishes were coated overnight with goat-anti human IgG and rICAM-1, or with poly-L-lysine (control). Jurkat cells (3.5*10^6^ cells/group) were washed, resuspended in CMF-PBS or LFA-1 activation buffer, and allowed to adhere to the coated Petri dishes for various time points (0–60 min) at 37°C. Cells were lysed in RIPA buffer (50 mM Tris-HCl, pH 8.0, 150 mM NaCl, 1 mM NaF, 1 mM NaVO_3_, 0.1% SDS, 0.1% sodium deoxycholate, 1% NP40) containing cOmplete mini protease inhibitor, and total protein was quantified by Bradford assay (BioTek, Winooski, VT). 20 μg total protein, boiled for 5 min in loading buffer, was subjected to SDS-PAGE. Membranes were stained with primary antibody (1 μg) overnight at 4°C followed by Alexa 680- or IR800-conjugated secondary antibody (1:10000) for 1 h at room temperature. Signals were detected with the Odyssey infrared imaging system (LI-COR, Lincoln, NE).

### Immunofluorescence assay

Glass coverslips were coated overnight with goat-anti human IgG and rICAM-1, or with poly-L-lysine (control). T cells (3.5*10^6^ cells/group) were washed and resuspended in CMF-PBS or LFA-1 activation buffer, and allowed to adhere to the coated coverslips for 45 min at 37°C. Cells were then fixed with paraformaldehyde (4%), permeabilized with Triton X-100 (0.1%), and blocked in normal goat serum (5%). Cells were sequentially stained with HuR antibody (1:100) overnight at 4°C followed by AlexaFluor-488 or cyanine 3-conjugated secondary antibody (1:1000) for 1 h at room temperature, then counterstained with DAPI (300 nM). Stained samples were fixed onto glass slides, visualized and photographed on an inverted Microphot fluorescent microscope (Nikon, Tokyo, Japan). HuR translocation was quantified as a ratio of the total area within the periphery of HuR staining divided by the nuclear area; 30 randomly selected cells per group were included per quantification.

### Co-immunoprecipitation assay

Jurkat cells (1*10^7^ cells/group) were washed and resuspended in IP buffer (25 mM HEPES, pH 8.0, 150 mM KCl, 840 μg/ml NaF, 1 mM DTT, 2.5 mM EDTA, 0.1% NP40) containing cOmplete mini protease inhibitor, and lysed by repeated syringing with a 1 ml syringe fitted with a 20 G needle. The lysates were cleared of insoluble debris by centrifugation at 10,621 g for 10 min at 4°C. For RNase digestion studies, lysates were additionally treated with RNase A (25 μg/10^6^ cells), or an equivalent volume of IP buffer (control), and incubated at 37°C for 30 min. The lysates were immunoprecipitated with HuR (2 μg/group), hnRNPC, H1 or K (1 μg/group) or MK2 (2.5 μg/group) antibody overnight at 4°C, followed by pull-down with protein A/G PLUS-agarose (20 μl) at 4°C for 4 h. The samples were then centrifuged at 3,824 g for 6 min at 4°C, washed three times with IP buffer, resuspended in RIPA buffer containing cOmplete mini protease inhibitor, and boiled for 5 min in loading buffer. The resulting immunoprecipitates were resolved by Western blot analysis.

### mRNA crosslinking assay

Petri dishes were coated overnight with goat-anti human IgG and rICAM-1, or with poly-L-lysine (control). Jurkat cells (1*10^7^ cells/group) were washed, resuspended in CMF-PBS or LFA-1 activation buffer, and allowed to adhere to the coated Petri dishes at 37°C for 45 min. The media was then removed, and the adherent cells on the plate were dislodged with trypsin-EDTA (0.05%) by incubating at 37°C for 5 min. Cells were then collected, washed and resuspended in CMF-PBS. Cells were fixed with formaldehyde (1%) for 10 min; the reaction was quenched with the addition of 250 mM glycine. Cells were resuspended in IP buffer, and lysed with two rounds of sonication at 70% efficiency using the sonic dismembrator 500 (Thermo Fisher). Lysates were subjected to immunoprecipitation with HuR, hnRNPC or isotype control (IgG1κ) antibody pre-conjugated with protein A/G PLUS-agarose at 4°C for 2 h. After incubating overnight at 4°C, the immunoprecipitate was harvested, the formaldehyde crosslinks were reversed by heating at 70°C for 45 min, and total RNA was isolated using the RNeasy mini kit and reverse transcribed. The resulting cDNA was subjected to real-time PCR analysis. Samples were run in triplicate and normalized to 18s rRNA to control for loading.

### Proteomic screening assay

Jurkat cells (2*10^7^ cells/group) were lysed, immunoprecipitated with HuR antibody, and the immunoprecipitate resolved by SDS-PAGE. Control samples were treated identically, but isotype control antibody (mouse IgG1k, clone MOPC-21) was used instead of HuR antibody. The gel was stained using SYPRO ruby protein gel stain, and imaged using the Typhoon 9410 scanner (GE Healthcare, Buckinghamshire, United Kingdom). Images were viewed using DeCyder 2D version 6.5 (GE Healthcare), and regions of interest were located using the Ettan Spot Picker robot (GE Healthcare) and excised. The lane was cut into the following ten molecular weight (kDa) ranges: 18–25, 23–25, 25–28, 28–31, 31–33, 33–35, 35–40, 40–43, 43–48 and 48–55. The gel bands were minced and subjected to *in situ* trypsin digestion. Each gel slice was washed in 250 μl acetonitrile (50%) for 5 min, then washed in 250 μl 50 mM ammonium bicarbonate/acetonitrile (50%) for 5 min. A final 30 min wash in 250 μl 10 mM ammonium bicarbonate/acetonitrile (50%) was performed prior to SpeedVac (Thermo Fisher) drying of the gel slices. 160 μl of trypsin (100 μg/ml) was added, and samples were incubated at 37°C for 16 h. 10 μl digest supernatant was analyzed via LC-MS/MS with the Waters/Micromass Q-Tof Ultima mass spectrometer equipped with the CapLC system (Waters, Milford, MA). 5 μl protein digest was directly injected onto a 100 μm x 150 mm Atlantis column (Waters) running at 500 nl/min. Initial HPLC conditions were: 95% buffer A (98% water, 2% acetonitrile, 0.1% acetic acid, 0.01% TFA), 5% buffer B (20% water, 80% acetonitrile, 0.09% acetic acid, 0.01% TFA) with the following linear gradient: 3 min, 5% buffer B; 43 min, 37% buffer B; 75 min, 75% buffer B; 85 min, 95% buffer B. Data-dependent acquisition was performed so that the mass spectrometer switched automatically from MS to MS/MS modes when the total ion current increased above the 1.5 counts/sec threshold set point. To optimize fragmentation, a collision energy ramp was set for the different mass sizes and charge states, giving preference to doubly or triply charged species for fragmentation.

### MS/MS analysis

MS/MS data were searched in-house using the Mascot algorithm [29] for un-interpreted MS/MS spectra after using Mascot Distiller (Matrix, Boston, MA) to generate Mascot-compatible files. Mascot Distiller combined sequential MS/MS scans from profile data with the same precursor ion. Charge states of +2 or +3 were preferentially located with a signal-to-noise ratio ≥ 1.2, and a peak list was generated for database searching in NCBInr. Using the Mascot algorithm, a protein was considered identified when Mascot listed it as a significant match and more than two peptides matched the same protein. Search parameters were: partial methionine oxidation and acrylamide modified cysteine, peptide tolerance ± 0.6 Da, MS/MS fragment tolerance ± 0.4 Da, peptide charge +2 or +3.

### Statistical analyses

In experiments involving more than two experimental groups, we determined whether the difference between the groups was statistically significant using one-way analysis of variance test and Bonferroni posttest. Otherwise, we used two-tailed student's *t* test. To perform all calculations we used GraphPad Prism software, version 6 (GraphPad Software, San Diego, CA).

## Supporting information

S1 Table Phosphopeptides identified from the isobaric tag for relative and absolute quantitation (iTRAQ) assay. (XLSX)

## Abbreviations

ARE: adenylate-uridylate (AU)-rich element
hnRNP: heterogeneous nuclear ribonucleoprotein
HuR: Hu protein R
ICAM-1: intercellular adhesion molecule-1
LFA-1: lymphocyte function-associated antigen-1
MAP: mitogen-activated protein
MK2: mitogen-activated protein kinase-activated protein kinase 2
MKK3: mitogen-activated protein kinase 3
pLL: poly-L-lysine
UTR: untranslated region
